# Th9 Cells in Peripheral Blood Increased in Patients with Immune-Related Pancytopenia

**DOI:** 10.1155/2020/6503539

**Published:** 2020-05-05

**Authors:** Qing Shao, Yangyang Wang, Zhaoyun Liu, Hui Liu, Yihao Wang, Yang Zhao, Lijuan Li, Rong Fu

**Affiliations:** Department of Hematology, Tianjin Medical University General Hospital, Tianjin 300052, China

## Abstract

**Background:**

Immune-related pancytopenia (IRP) is a kind of autoimmune disease mediated by autoantibodies in bone marrow. T helper 9 (Th9) cell is a new subset of T cell discovered recently, which mainly expresses cytokine interleukin-9 (IL-9) to exert immune function. Th9 cells are associated with a variety of inflammatory diseases, but the role of Th9 cells in IRP remains unclear.

**Methods:**

Fifty patients with IRP and 20 healthy controls were enrolled. The percentage of Th9 cells was detected by flow cytometry (FCM) and ELISA. CD4^+^ lymphocytes were sorted by magnetic beads, and the mRNA expression levels of Th9 cells related transcription factors PU.1 and BATF were detected by RT-PCR.

**Results:**

The percentage of Th9 cells in CD3^+^CD4^+^ cells was 2.73 ± 1.96% in the untreated group, which was significantly higher than those in the remission group (1.21 ± 0.86%) (*p* < 0.01) and the control group (0.68 ± 0.40%) (*p* < 0.001). And that in the remission group was significantly higher than that in the control group (*p* < 0.05). The level of IL-9 in the untreated group was 183.91 ± 112.42 pg/mL, which was significantly higher than that in the remission group (105.96 ± 64.79 pg/mL) (*p* < 0.01) and control group (56.03 ± 14.49 pg/mL) (*p* < 0.001). That in the remission group was also significantly higher than that in the control group (*p* < 0.01). They were negatively correlated with hemoglobin, red blood cell, white blood cell, and platelet counts and positively correlated with the percentage of CD19^+^B cells and CD5^+^CD19^+^/CD19^+^B cells, respectively. The mRNA expression levels of PU.1 and BATF in IRP patients were higher than those in controls (*p* < 0.05).

**Conclusions:**

The percentage of Th9 cells in the peripheral blood and the level of IL-9 in the serum of patients with IRP were increased, which was related to the severity of the disease.

## 1. Introduction

Immune-related pancytopenia (IRP) is a bone marrow failure disease mediated by autoantibodies [1]. The clinical manifestations are as follows: (1) there is pancytopenia with a high or normal proportion of reticulocytes and neutrophils; (2) the proportion of nucleated erythrocyte in bone marrow is normal or elevated, and “erythropoietic islands” can often be seen under a microscope [2]; (3) known hematologic diseases, including aplastic anemia (AA), hemolytic anemia, megaloblastic anemia, and myelodysplastic syndrome (MDS), are excluded; (4) high dosage of immunoglobulin, glucocorticoid, and other immunosuppressive agents is effective. Our research group had detected autoantibodies on the membrane of bone marrow hematopoietic cells by a bone marrow mononuclear cell- (BMMNC-) Coombs test or FCM. It was confirmed that the disease was mainly caused by abnormal humoral immunity [3].

Autoantigens in IRP were investigated by membrane protein extraction from BM hemopoietic cells and BM supernatant from IRP patients. This study identified that a G-protein-coupled receptor 156 variant and chain P, a crystal structure of the cytoplasmic domain of human erythrocyte band-3 protein, were autoantigens in IRP [3]. In addition, our team also screened new autoantigens in IRP by serological analysis of recombinant cDNA expression libraries and compared anti-UQCR10 (ubiquinol-cytochrome c reductase, complex III subunit X) antibody levels between IRP and normal controls detected by immunoblotting. It was found that UQCR10 may be one of the autoantigens involved in IRP formation [4].

We had also conducted a preliminary study on the humoral immune status in patients with IRP. The results showed that the quantity and function of CD5^+^B lymphocytes increased in IRP patients [5, 6]. And the autoantibodies may destroy hematopoietic cells through three ways: autoantibodies destroy hematopoietic cells through complement activation [7]; some autoantibodies (IgG) block EPOR on nucleated erythrocyte membrane, which resulted in blocked signal of hematopoietic factors in bone marrow [8]; autoantibody IgG activates macrophages to phagocytize and destroy bone marrow hematopoietic cell antibody [9, 10]. The proportion balance of Th1/Th2 cells shifted to the Th2 direction [11]. The quantity and function of Th17 cells, which was named follicular helper T cells, increased [12–14], while Tregs and NK cells decreased in the IRP patients [14, 15]. The proportion balance of pDC/mDC cells shifted to the pDC direction [16]. In conclusion, IRP has a complex immune regulation imbalance.

Th9 cell (helper T cell 9) is recently discovered as a new type of helper T cells, which is characterized by secreting IL-9 [17]. Th9 cell development requires coinduction of transforming growth factor-beta (TGF-*β*) and interleukin-4 (IL-4) [18]. Spi-1 proto oncogene (PU.1) and basic leucine zipper ATF-like transcription factor (BATF) are important transcription factors in the secretion of IL-9 by Th9 cells, which were activated by different signaling pathways [19–21]. Th9 cells are related to a variety of inflammatory diseases [22]. The purpose of this study is to detect the quantity and function of Th9 cells, to explore the role of Th9 cells in IRP, and to provide theoretical basis for the diagnosis and treatment of IRP.

## 2. Methods

### 2.1. Patients and Methods

A total of 50 patients with IRP were enrolled in this study, including 30 untreated patients and 20 patients in remission, who were all inpatients in the Department of Hematology, Tianjin Medical University General Hospital (Tianjin, China), from July 2017 to January 2018. The characteristics of the patients are shown in Table 1.

All the patients received corticosteroids (prednisone, 0.5 mg/kg/day) and cyclosporine (CsA) (3 mg/kg/day) as immunosuppressive therapy, and some received high-dose IVIg (0.4 g/kg/day for 5 days; Chengdu Institute of Biological Products, Sichuan, China) if they depend on blood transfusion. Complete blood count (CBC) and bone marrow (BM) examination were performed regularly. The therapeutic effect was determined according to response criteria of aplastic anemia (AA) [23], and the median follow-up time was 12 months (range, 3-21 months).

A total of 20 healthy donors (11 females and 9 males; median age, 25 years; range, 20-32 years) were selected as normal controls. This study was approved by the Ethics Committee of Tianjin Medical University and published with the informed consent of patients.

### 2.2. Enzyme-Linked Immunosorbent Assay (ELISA)

The serum level of IL-9 in the patients with IRP and the control group was measured by ELISA (human, SEA081Hu; USCN LIFE, Wuhan, China). According to the protocol, the diluted standard was added into six holes of a 96-well plate, with a minimum detectable concentration of 6.1 pg/mL and a maximum concentration of 1000 pg/mL. The eight holes were used to make a standard curve. The remaining holes were added with 100 *μ*L peripheral blood serum, covered with transparent membrane and incubated in a 37°C incubator for one hour. Discard the liquid in the hole; then add the detection solution A 100 *μ*L into each hole, and place it in the incubator of carbon dioxide at 37°C for 1 hour. After washing the discarded liquid with 350 *μ*L of detergent for 3 times, solution B 100 *μ*L was added to each hole in the incubator for 30 minutes. The plate was washed five times, TMB substrate was subsequently added to each well, and the samples were incubated in the dark at 37°C for 20 minutes. Finally, a terminating solution was added, and the optical density was obtained at 450 nm within 15 minutes.

### 2.3. MACS

Peripheral blood mononuclear cells (PBMCs) were isolated by Ficoll-Hypaque density gradient centrifugation. The diluted blood was slowly added to the equal volume Ficoll-Hypaque solution and then centrifuged by 500g for 20 minutes at 4°C. Intermediate white flocculent cells are the mononuclear cells we need. Every 10^7^ PBMCs are resuspended in 80 *μ*L buffer. CD4^+^ T lymphocytes were purified using CD4^+^ T cell isolation kit (Miltenyi Biotec GmbH, Bergisch Gladbach, Germany) according to the manufacturer's protocol. Then, 20 *μ*L CD4 biotin-antibody was added. The cells were incubated at 4°C for 15 minutes in the dark, washed with 1 mL PBS, and then suspended in 500 *μ*L buffer. The MS column is placed on the MACS separator. After washing with 1 mL buffer, the cells were added to the column. The labeled cells were collected on the column. The unlabeled cells were collected on one tube. By firmly pushing the plunger into the column with 1 mL buffer, the magnetic labeled cells were immediately washed out. Finally, CD4^+^ cells were collected on another tube. Cell suspension was evenly divided into two tubes: one was incubated with CD4-FITC (130092358, 1 : 10; Miltenyi Biotec GmbH) in darkness for 15 minutes as the experimental tube, and another one was incubated with IgG_1_-FITC (130098847, 1 : 10; Miltenyi Biotec GmbH) in the darkness for 15 minutes to stain as a negative control. At last, the purity was determined by FCM (BD Biosciences).

### 2.4. Reverse Transcription Quantitative Polymerase Chain Reaction (RT-PCR)

The relative expression levels of Th9 cell-related transcription factors PU.1 and BATF mRNA were detected by RT-PCR. The primer sequences used in this study are shown in Table 2. Total RNA was isolated from CD4^+^ T lymphocytes by TRIzol reagent (Takara Biotechnology Co. Ltd., Dalian, China). PrimeScript reverse transcription kit (Takara Biotechnology Co. Ltd.) was used to convert 1 *μ*g RNA into cDNA at 37°C for 15 minutes and then 5 seconds at 85°C for 1 cycle. PCR was performed in 25 *μ*L reaction volume containing 12.5 *μ*L SYBR-Green (Takara Biotechnology Co. Ltd.). All primer sequences are listed in Table 2. The thermal cycle curves were 95°C for 5 seconds and 60°C for 45 seconds for 45 cycles. A quantitative cycling (Cq) method was used to calculate the relative quantity of target gene expression.

### 2.5. FCM Analysis

PBMC was incubated with 10 *μ*L and 1 *μ*g/mL PMA (Shanghai, Beijing Institute of Biotechnology, China), 8 *μ*L and 0.5 mg/mL BFA (Beijing Institute of Biotechnology), and 8 *μ*L and 50 *μ*g/mL ionomycin (Beijing Institute of Biotechnology) at 37°C for 4 hours. Second, CD3-APC and CD4-FITC antibodies were incubated for 15 minutes at 4°C in the dark. Cells after hemolysis were immobilized and perforated on the cell membrane model by BD Pharmingen. The cells were divided into two groups: the experimental group and the control group. Cells stained with IL-9-PE (human, 560807, BD Biosciences) were used as the experimental group, while cells stained with IgG_1_-PE (130098845, 1 : 10; Miltenyi Biotec GmbH) were used as the negative control group. The cells were incubated at 4°C for 15 minutes in the dark, washed with 1 mL PBS, and then suspended in 300 *μ*L PBS. Meanwhile, autoantibodies on the membrane of bone marrow hematopoietic cells were detected by FCM (percentage of CD15^+^IgG^+^ cells and GlycoA^+^IgM^+^ cells) [3]. At least 10^4^-10^5^ cells were collected and analyzed by FACS (BD Biosciences) and Cell Query Software version 6.0.

### 2.6. Statistical Analysis

Results were analysed with SPSS 21.0. Continuous variables were expressed as the mean ± standard error of the mean. The significance of the difference was assessed by one-way ANOVA, and then the least significant difference test of homogeneous variance or the Tamhane test of nonhomogeneous variance was used for multiple special postcomparison. The correlation between patient characteristics was tested by Spearman's correlation methods. *p* < 0.05 was considered statistically significant.

## 3. Results

### 3.1. The Percentage of Th9 Cells in IRP Patients Was Significantly Increased and Correlated with Clinical Data

**Figure 1:**
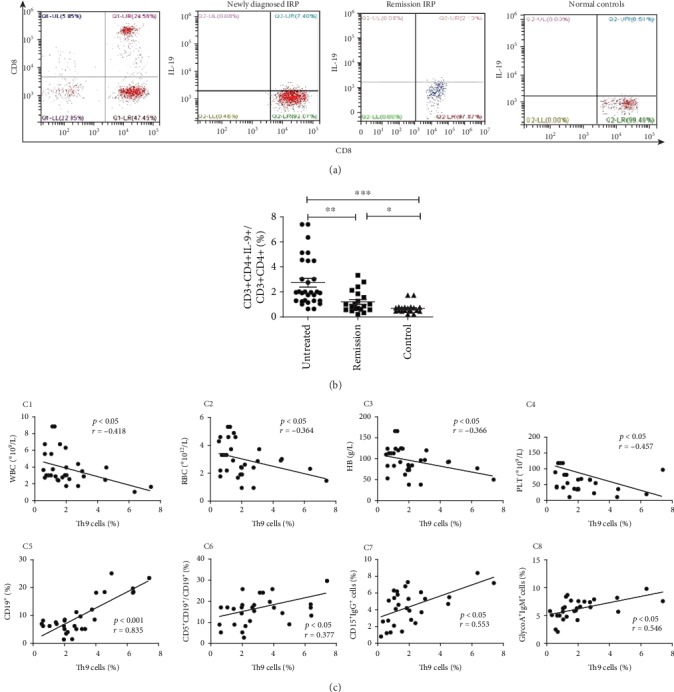
The percentage of Th9 in IRP patients and the correlation with clinical data. (a) The percentage of Th9 cells detected by FCM. (b) The percentage of Th9 among three groups was compared by statistical analysis. (c) Correlation analysis of the percentage Th9 cells with clinical indicators. (C1–C4) The percentage of Th9 was negatively correlated with WBC count, RBC, Hb, and PLT count. (C5 and C6) The percentage of Th9 was positively correlated with the percentage of CD19^+^ cells and CD5^+^CD19^+^/CD19^+^. (C7 and C8) The percentage of Th9 was positively correlated with the percentage of CD15^+^IgG^+^ cells and GlycoA^+^IgM^+^ cells.

The percentage of Th9 cells in CD3^+^CD4^+^ cells was 2.73 ± 1.96% in the untreated group, which was significantly higher than those in the remission group (1.21 ± 0.86%) (*p* < 0.01) and the control group (0.68 ± 0.40%) (*p*<0.001) (Figures 1(a) and 1(b)). And that in the remission group was significantly higher than that in the control group (*p* < 0.05).

The percentage of Th9 cells was negatively correlated with Hb (*p* < 0.05, *r* = −0.366), RBC (*p* < 0.05, *r* = −0.364), PLT (*p* < 0.05, *r* = −0.457), and WBC (*p* < 0.05, *r* = −0.418) and positively correlated with the percentage of CD5^+^CD19^+^/CD19^+^ cells (*p* < 0.05, *r* = 0.377) and the percentage of CD19^+^ cells (*p* < 0.001, *r* = 0.835). The percentage of Th9 cells was positively correlated with the percentage of CD15^+^IgG^+^ cells (*p* < 0.05, *r* = 0.553) and GlycoA^+^IgM^+^ cells (*p* < 0.05, *r* = 0.546) (Figure 1(c)).

### 3.2. The Serum Level of IL-9 in IRP Patients Was Significantly Increased and Correlated with Clinical Data

**Figure 2:**
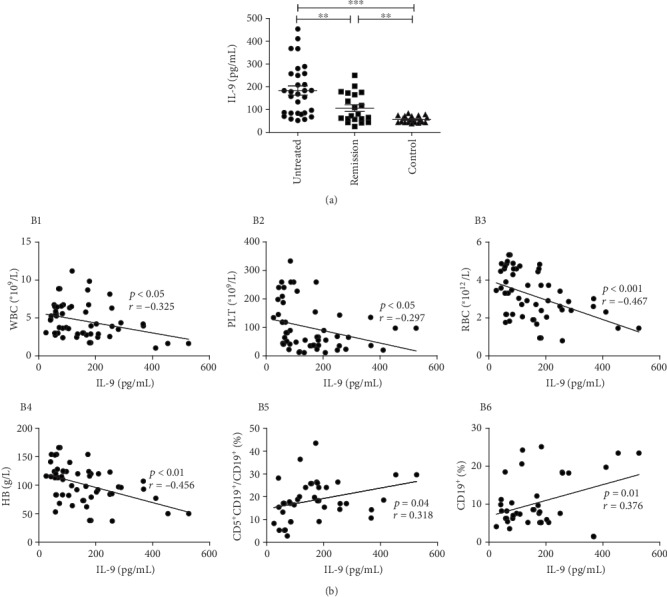
The serum level of IL-9 in IRP patients and the correlation with clinical data. (a) Serum levels of IL-9 in the untreated IRP patients, the remission patients and the volunteers were treasured by ELISA. (b) Correlation analysis of level of IL-9 with clinical indicators. (B1–B4) The serum level of IL-9 was negatively correlated with WBC count, RBC, Hb, and PLT count. (B5 and B6) The serum level of IL-9 was positively correlated with the percentage of CD19^+^ cells and CD5^+^CD19^+^/CD19^+^.

The level of IL-9 in the untreated group was 183.91 ± 112.42 pg/mL, which was significantly higher than those in the remission group (105.96 ± 64.79 pg/mL) (*p* < 0.01) and control group (56.03 ± 14.49 pg/mL) (*p* < 0.001). And that in the remission group was also significantly higher than that in the control group (*p* < 0.01) (Figure 2(a)).

The analysis between serum levels of IL-9 and clinical indicators showed that the level of IL-9 was negatively correlated with Hb (*p* < 0.01, *r* = −0.457), RBC (*p* < 0.05, *r* = −0.467), PLT (*p* < 0.05, *r* = −0.297), and WBC (*p* < 0.05, *r* = −0.227). The level of IL-9 was positively correlated with the percentage of CD5^+^CD19^+^/CD19^+^ cells (*p* < 0.05, *r* = 0.376) and the percentage of CD19^+^ cells (*p* < 0.05, *r* = 0.318) (Figure 2(b)).

### 3.3. The Relative Expressions of Th9 Cell-Related Transcription Factor PU.1 and BATF mRNA Were Significantly Increased in Patients with IRP

**Figure 3:**
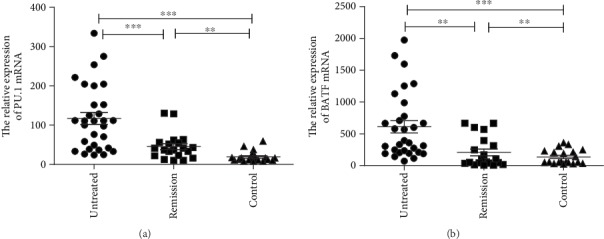
(a) The relative expression of PU.1 mRNA in CD4^+^ T cells was detected by RCR. (b) The relative expression of BATF mRNA in CD4^+^ T cells was detected by RCR.

The relative expression of PU.1 mRNA was 117.30 ± 82.33 in the untreated group, 45.46 ± 33.22 in the remission group, and 19.07 ± 13.56 in the control group. The relative expression of PU.1 mRNA in the untreated group was significantly higher than those in the control group (*p* < 0.01) and the remission group (*p* < 0.001). And that in the remission group was significantly higher than that in the control group (*p* < 0.01) (Figure 3(a)).

The relative expression of BATF mRNA was 608.57 ± 517.68 in the untreated group, 238.89 ± 206.80 in the remission group, and 134.71 ± 113.31 in the control group. That in the untreated group was significantly higher than that in the remission group (*p* < 0.01). There was no significant difference in the relative expression of BATF mRNA between the remission group and the control group (*p* > 0.05) (Figure 3(b)).

## 4. Discussion

IRP is a kind of hemocytopenia caused by increased destruction or functional inhibition of bone marrow hematopoietic cells mediated by autoantibodies. It has a good response to immunosuppressive therapy. Our research group has carried out a preliminary study on the quantity and function of various immune cells in the IRP patients. Th9 cell is a new type of the CD4^+^ T helper cell subgroup, which has important regulation on the immune mechanism of the human body. It has been reported that Th9 cells are related to autoimmune diseases by secreting IL-9, such as systemic lupus erythematosus (SLE) [24], allergic asthma [25], ulcerative colitis (UC) [26, 27], and rheumatoid arthritis (RA) [28]. IL-9 binds to IL-9R, phosphorylating tyrosine tyr407 on the IL-9R*α* chain, activating JAK1, and on another *γ* chain, activating JAK3. Signal transduction activates STATs, triggers a series of related gene expression, and exerts corresponding biological effects [29].

The humoral immunity and cellular immunity of normal people are in the dynamic balance of physiological state, while the humoral immunity and cellular immunity of the IRP patients are in the dynamic balance of pathological state. The proportion of Th1/Th2 cells in IRP patients shifted to Th2 cells, which increased the proportion of Th2 cells and the secretion of IL-4 and IL-10 cytokines, enhanced the positive regulation of B cells, and promoted the production of autoantibodies [5, 14]. Bone marrow hematopoietic stem cells/progenitor cells were inhibited or destroyed, resulting in the decrease of blood cells.

A previous study had proven that IL-9 plays an important role in B cell maturation and function [30]. Petit Frere et al. studied that the enhanced effect of IL-9 on IL-4-induced IgE and IgG_1_ released by mouse B lymphocytes was stimulated by lipopolysaccharide (LPS), suggesting the role of IL-9 in B cell stimulation of allergy and autoimmune response [31]. In our study, the serum level of IL-9 in IRP patients was increased. And the level of IL-9 was positively correlated with the percentage of CD19^+^ cells and the percentage of CD5^+^CD19^+^/CD19^+^ cells. Therefore, it is speculated that IL-9 may enhance the secretion of auto-antibodies by activating B lymphocyte in the patients, thus aggravating the progress of the IRP disease.

In this study, the percentage of Th9 cells in all CD3^+^CD4^+^ cells in IRP patients was increased. The relative expression of Th9 cell-related transcription factors PU.1, BATF mRNAs were also increased. The percentage of Th9 cells was positively correlated with the percentage of CD19^+^ cells and the percentage of CD5^+^CD19^+^/CD19^+^ cells and negatively correlated with Hb, RBC, PLT, and WBC. So it is speculated that with the increasing of the quantity of Th9 cells, the activation of B cell function may further promote the occurrence and development of diseases.

It is notable that Th1, Th17, and Th9 cells all have the capacity to produce IL-9. Th9 cells and IL-9 contribute to inflammatory responses in SLE patients, and IL-9 is an important source of inflammatory cytokines. IL-9 can promote B cell proliferation and immunoglobulin production, which can be blocked by the inhibition of STAT3. Treatment with neutralizing anti-IL-9 antibody in vivo decreased serum anti-dsDNA-antibody titers and alleviated lupus nephritis in MRL/lpr mice, which suggests that IL-9 is a potential therapeutic target for SLE [32]. Conversely, IL-9-producing CD4^+^ T cells also promote the suppressive functions of Tregs. As an anti-inflammatory cytokine, which protects against external danger signals, IL-9 produced by Th17 can facilitate the function of Tregs. In this case, the immunosuppressive activity of Tregs will lead to a decrease in effector T cells and impact defenses against foreign organisms or substances [22].

Although the research on Th9 cells is not comprehensive, evidence is accumulated to demonstrate a role of IL-9 and Th9 cells in the pathogenesis of a spectrum of autoimmune diseases. Neutralization of anti-IL-9 antibody reduces the titer of autoantibody and reduces the inflammatory response in vivo, suggesting that IL-9 may be a therapeutic target for autoimmune diseases. At the same time, further researches are needed to clarify the interactions between IL-9 and other parts of the immune system.

## Figures and Tables

**Table 1:** Characteristics of patients with Immune-related Pancytopenia.

	The untreated IRP patients	The remission IRP patients
*N*	30	20
Female/male	18 : 12	13 : 07
Age (median, range)	36 (13-81)	35 (9-71)
Hemoglobin (g/L) (range)	92.5 (38-166)	118 (37-154)
Platelets (∗10^9^), (range)	60 (11-333)	139 (22-259)
Erythrocytes (∗10^9^), (range)	3.02 (1.04-8.85)	5.27 (1.64-9.84)
Leucocytes (∗10^9^), (range)	2.89 (0.95-5.33)	3.81 (0.8-4.98)
Reticulocytes (%) (range)	2.42 (0.39-5.68)	2.15 (1.23-21.47)
Neutrophils (%) (range)	50.5 (20.3-91.3)	58.05 (3.98-90.5)
Lymphocyte (%) (range)	41.3 (2.2-61.4)	31.7 (5.8-53.3)
Abnormal chromosome	0	0

**Table 2:** The primer sequences used in this study.

Gene	Sense (5′-3′)	Antisense (5′-3′)
PU.1	GATCCGCCTGTACCAGTTCC	CTCCTTGTGCTTGGACGAGA
BATF	CAGACACAGAAGGCCGACAC	GTTCAGCACCAGCGTGAAGT
*β*-Actin	TGGACATCCGCAAAGACCTGT	CACACGGAGTACTTGCGCTCA

PU.1: Spi-1 proto oncogene; BATF: basic leucine zipper ATF-like transcription factor.

## Data Availability

The data used to support the findings of this study are available from the corresponding author upon request.
